# Polydimethylsiloxane Organic–Inorganic Composite Drug Reservoir with Gliclazide

**DOI:** 10.3390/ijms25073991

**Published:** 2024-04-03

**Authors:** Ahmed Gedawy, Hani Al-Salami, Crispin R. Dass

**Affiliations:** 1 Curtin Medical School, Curtin University, Bentley 6102, Australia; ahmed.gedawy@postgrad.curtin.edu.au (A.G.); hani.al-salami@curtin.edu.au (H.A.-S.); 2 Curtin Health Innovation Research Institute, Curtin University, Bentley 6102, Australia

**Keywords:** gliclazide, composite bead, chitosan, PDMS, myoblast, skeletal muscle

## Abstract

A novel organic–inorganic gliclazide-loaded composite bead was developed by an ionic gelation process using acidified CaCl_2_, chitosan and tetraethylorthosilicate (TEOS) as a crosslinker. The beads were manufactured by crosslinking an inorganic silicone elastomer (-OH terminated polydimethylsiloxane, PDMS) with TEOS at different ratios before grafting onto an organic backbone (Na-alginate) using a 3^2^ factorial experimental design. Gliclazide’s encapsulation efficiency (EE%) and drug release over 8 h (% DR 8 h) were set as dependent responses for the optimisation of a pharmaceutical formula (herein referred to as ‘G op’) by response surface methodology. EE % and %DR 8 h of G op were 93.48% ± 0.19 and 70.29% ± 0.18, respectively. G op exhibited a controlled release of gliclazide that follows the Korsmeyer–Peppas kinetic model (R^2^ = 0.95) with super case II transport and pH-dependent swelling behaviour. In vitro testing of G op showed 92.17% ± 1.18 cell viability upon testing on C2C12 myoblasts, indicating the compatibility of this novel biomaterial platform with skeletal muscle drug delivery.

## 1. Introduction

Polydimethylsiloxanes (PDMSs) are an ecofriendly class of polymers that degrade into non-toxic byproducts (silicic acid and CO_2_) [1]. Due to their biocompatibility and drug permeability characteristics, silicone elastomers are employed in many marketed pressure-sensitive adhesives, body inserts and implants [2]. Two types (reservoir and matrix) were identified for silicone drug delivery vehicles [3]. Channelling for hydrophilic drugs was suggested as the release mechanism through silicone elastomers, while diffusion followed by release was described for lipophilic substrates [3]. Implantable drug delivery systems are employed in chronic diseases to obtain a steady state-controlled drug release that bypasses the first pass effect (hepatic metabolism), and they often achieve higher local therapeutic concentration in a targeted tissue, minimising systemic side effects [4,5,6]. Lipophilic and low molecular weight drugs were reported to easily diffuse through silicone elastomers [7,8]. Due to their solubility in the lipophilic silicone elastomers and their low molecular weight, steroids were released from silicone carriers to allow for rapid molecular diffusion [9]. Norplant^®^ is a levonorgestrel-loaded PDMS implant used for human contraception [10,11]. Compudose^®^ is a silicone-based subdermal ear implant for veterinary controlled delivery of oestradiol to enhance growth [12,13]. Crestar^®^ is another veterinary silicone-based ear implant that uses norgestomet for oestrous synchronisation [14].

Modification of PDMS is often required to achieve a suitable implantable elastomeric drug delivery system [9,15,16]. Incorporation of a hydrogel (hydrophilic polymer) within the PDMS structure has been reported as one of the techniques to achieve that goal [9] by fostering a biphasic composite with good mechanical properties and controlled water uptake characteristics [17,18]. Alginate [19,20,21] and chitosan [22,23] are two natural hydrogel biopolymers that have also been used in tissue engineering, implants and tissue regeneration in various body parts [6]. Alginate encapsulation has been widely reported as a pharmaceutical drug delivery technique that is able to sustain and target the drug’s release [24,25,26]. The electrostatic interaction between the alginate (a polyanion) and chitosan (a polycation) in the presence of Ca^++^ ions employing ionic gelation was also reported to produce sustained release microcapsules [27,28]. In fact, this polyelectrolyte ionic gelation interaction in the presence of Ca^++^ ions was discussed in detail by Gaserod et al. [29,30]. The formed alginate/chitosan complex coacervate is irreversible and resulted in the production of mechanically strong beads [29,30]. The authors concluded that a more porous gelled network is formed in the existence of Ca^++^ ions with chitosan, where both calcium and chitosan compete for binding to alginate [29,30]. Tetraethyl orthosilicate (TEOS), a silica precursor alkoxysilane that has been extensively studied in the crosslinking of PDMS [31,32,33], was also reported to form a silica–alginate hybrid composite [34,35] either by the interaction with alginate [35] or coating the crosslinked Ca-alginate surface [34]. TEOS, on the other hand, was reported to interact non-covalently with chitosan to form a pH-sensitive interpenetrative polymeric network (either by van der Waals attraction or through H-bonding) [36,37,38,39,40,41,42]. This physical crosslinking between the entangled polymeric chains of chitosan and TEOS produced a unique film that was mechanically strong due to TEOS and flexible due to chitosan and was investigated in modifying drug release [36,37,38,39,40,41,42].

Insulin resistance, impaired glucose homeostasis and the inadequate glucose uptake of diabetic body tissues are well-documented diabetes-associated symptoms [43,44,45]. Among the strategies to reverse these metabolic defects is the enhancement of glucose utilisation by the largest body organ/tissue, the skeletal muscles (40% of the body weight) [43,44,45,46], a pharmacological effect mediated through glucose transporters GLUT1 and GLUT4 [43,44,45]. Sulfonylureas such as gliclazide were reported to enhance insulin signalling and have a direct action on skeletal muscle glucose disposal through a GLUT4-mediated effect [47,48,49,50]. Gliclazide is usually used as a substitute for metformin-intolerant type 2 diabetic patients [51,52,53]. However, hypoglycaemia associated with its oral administration, especially at high doses (up to 320 mg/day in the case of gliclazide), constitute a major therapeutic limitation [51,52,53]. Gliclazide has a 10–12 h half-life [53] and is subjected to extensive hepatic metabolism following its oral administration that results in inactive urinary excreted metabolites [51,52,53]. These pharmacokinetic properties of gliclazide have driven formulators to modify its release characteristics from oral dosage forms to achieve the therapeutic targets with fewer side effects [54]. To this end, gliclazide seems to be a good candidate for inclusion in an implantable dosage form due to its physicochemical similarity to the reported implanted steroids in terms of lipophilicity and low molecular weight. An implanted long-term sulfonylurea (glibenclamide) was investigated in one study [55]. The authors highlighted the beneficial effect of the pulsatile release of glibenclamide from the proprietary implanted beads on reversing the β-cell insulin secretory malfunction associated with the chronic use of sulfonylureas and suggested the use of a short-acting analogue [55].

This study aims to develop, optimise and characterise a novel gliclazide-loaded organic–inorganic composite bead via the interaction of a PDMS-grafted alginate with chitosan/TEOS in the presence of Ca^++^ ions using an ionic gelation technique. These novel antidiabetic beads were optimised using response surface methodology for higher gliclazide content (model drug) and to obtain a steady state gliclazide release. In vitro cytotoxicity testing of these novel beads on a murine myoblast cell line (C2C12) was also performed to evaluate potential cytotoxicity to skeletal muscle cells.

## 2. Results

### 2.1. Rheological Studies

**Figure 1:**
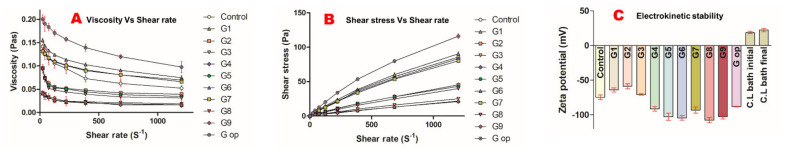
Rheological properties of polymeric dispersion (**A**,**B**). Electrokinetic stability (**C**).

Increasing the shear rate applied on the polymeric dispersion of all formulations resulted in decreased viscosities in a non-linear manner (Figure 1A). The graphical presentation of shear stress versus shear rate (Figure 1B) verifies the non-Newtonian characteristics of this polymeric platform.

### 2.2. Electrochemical Stability

The G2 formula had the lowest negative surface charge of −58.77 mV ± 3.71, G8 had the highest negative surface charge of −107.67 mV ± 3.79, while the optimised formula had a zeta potential of −88.17 mV ± 0.6 (Figure 1C). In contrast, the crosslinking bath (CL bath) had an initial positive surface charge of 18.97 mV ± 1.72, which was slightly increased at the end of the encapsulation process to 22.57 mV ± 2.59 (Figure 1C).

### 2.3. Characteristics of the Silicone Latex Employed in the Formulation of the Optimised Formula

SDS-emulsified PDMS latex used in the formulation of G op had a mean particle size of 169.5 nm ± 1.49 (polydispersity index of 0.21) and a zeta potential of −102.67 mV ± 2.08.

### 2.4. Compressibility and Flow Properties of Formulated Beads

All beads had good flow characteristics as per USP 37. The blank control and G op had Carr’s indices of 11.07 ± 1.28 and 12.21 ± 0.31, respectively, while Carr’s index for G1–G9 was in the range of 11.05 ± 1.59 to 12.29 ± 1.21. Hausner’s ratios for all formulations were in the range of 1.12 ± 0.016 to 1.14 ± 0.015 (Table 1).

### 2.5. Bead Content of Gliclazide and Entrapment Efficiency (EE%) and Response Surface Methodology Results for Gliclazide’s EE%

**Figure 2:**
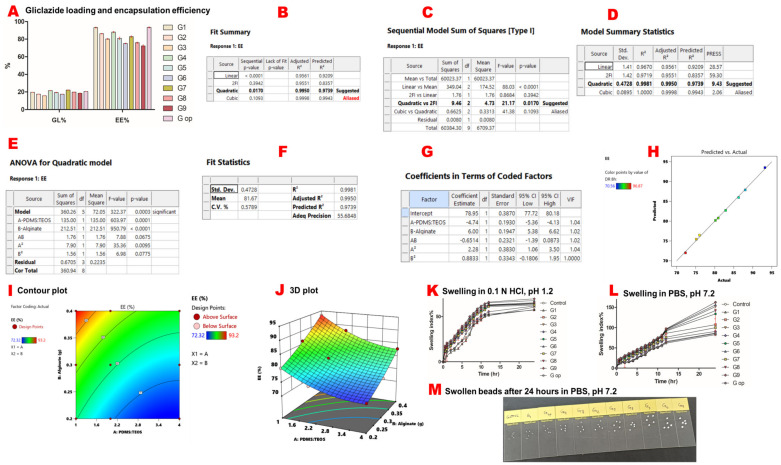
Gliclazide content, response surface methodology results of gliclazide’s encapsulation efficiency and swelling studies. Gliclazide loading (GL%) and encapsulation efficiency (EE%) (**A**). The quadratic mathematical model suggested by Expert Design^®^ for gliclazide’s EE (**B**). The quadratic versus two-factor interaction models (**C**). The quadratic model summary statistics (**D**). ANOVA results for the quadratic model (**E**). Precision, fit statistics and R^2^ of the suggested quadratic model (**F**). Coefficients of the polynomial equation that governs gliclazide’s EE% in terms of coded factors (**G**). Predicted values suggested by Expert Design^®^ versus actual experimental results (**H**). The two-dimensional contour plot of EE% (**I**). The three-dimensional presentation of EE% (**J**). Swelling in 0.1 N HCl, pH 1.2 (**K**). Swelling in PBS, pH 7.2 (**L**). Image of swollen beads after 24 h in PBS, pH 7.2 (**M**).

G3 exhibited the lowest gliclazide content of 15.71% ± 0.035, while G7 had the highest gliclazide loading (22.1% ± 0.087) of the nine experimental beads. The entrapment efficiency ranged between 72.32% ± 0.465 for G9 and 93.20% ± 0.24 for G1. G op had a gliclazide loading of 20.56% ± 0.04 and an entrapment efficiency of 93.48% ± 0.19 (Table 1) (Figure 2A).

A quadratic mathematical model was suggested for gliclazide’s EE% from the experimental factorial design of the study (Figure 2B–D). The significance of this model was indicated by a *p*-value of 0.0003 which was validated by an F-value of 322.37 (Figure 2E). Moreover, a small difference (<0.2) between predicted (R^2^ = 0.9739) and adjusted (R^2^= 0.9950) values, along with a high precision of 55.685 (>4 is statistically desirable), verified the accuracy and significance of the model (Figure 2F). Model parameters are denoted as follows: A: PDMS:TEOS ratio, B: alginate content, AB: the interaction between the PDMS:TEOS ratio and alginate content, A^2^: (PDMS:TEOS ratio)^2^, B^2^: (alginate content)^2^ (Figure 2E,G).

In this model, A, B and A^2^ were significant model terms in determining the entrapped gliclazide (EE%) within the formulated beads (*p* < 0.05). However, neither AB nor B^2^ were significant terms (*p* > 0.05) (Figure 2E). The relation between the actual and predicted values is presented in Figure 2H. The polynomial equation that governs EE% is EE% = 78.95 − 4.74 A + 6 B − 0.6514 AB + 2.28 A^2^ + 0.8833 B^2^. Since AB and B^2^ are insignificant model terms (*p*-value > 0.05), the equation can be simplified as follows: EE% = 78.9 − 4.74 A + 6 B + 2.28 A^2^.

The two-dimensional contour plot between A and B indicated the non-linear relationship between these two variables in determining gliclazide’s EE% (Figure 2I), while the three-dimensional plot revealed that decreasing the PDMS:TEOS ratio and increasing the alginate content improved the gliclazide EE% (Figure 2J).

### 2.6. Microcapsules’ Mechanical Integrity

All formulated beads were intact (100% integrity) after mechanical shaking in both test media for 24 h. However, microcapsules exhibited more swelling in PBS than in saline. 

### 2.7. Swelling Behaviour

All microcapsules showed more reduced swelling characteristics in acidic media than in PBS. G9 had the highest swelling index in HCl of 69.67% ± 0.58, while G op had the lowest swelling index in HCl of 57.33% ± 0.58 (Figure 2K). In PBS, the blank control had the lowest swelling index of 80.67% ± 3.1, while G9 exhibited the highest swelling index of 162.67% ± 2.5 (Figure 2L,M).

### 2.8. In Vitro Gliclazide Release, Mathematical Models for Gliclazide Release and Response Surface Methodology Results of Cumulative Gliclazide Release over 8 h (DR 8 h)

**Figure 3:**
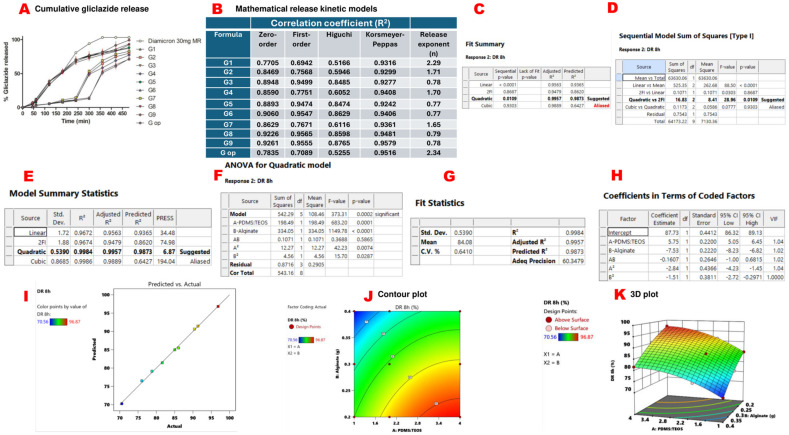
In vitro gliclazide release and response surface methodology results of gliclazide release over 8 h (DR 8 h). Cumulative gliclazide release from different beads compared to marketed gliclazide modified release formula (Diamicron^®^ 30 mg MR) (**A**). Mathematical release kinetic models (**B**). Quadratic mathematical model suggested by Expert Design^®^ for gliclazide %DR 8 h (**C**). The quadratic versus two-factor interaction models (**D**). Quadratic model summary statistics (**E**). ANOVA results for the quadratic model (**F**). Precision, fit statistics and R^2^ of the suggested quadratic model (**G**). Coefficients of the polynomial equation that governs gliclazide %DR 8 h in terms of coded factors (**H**). Predicted values suggested by Expert Design^®^ versus actual experimental results (**I**). The two-dimensional contour plot of %DR 8 h (**J**). The three-dimensional presentation of %DR 8 h (**K**).

All formulated microcapsules were able to sustain gliclazide release over 8 h compared to the marketed sustained release gliclazide tablet (Diamicron^®^ 30 mg MR). G9 released 96.87% ± 0.16 of the entrapped gliclazide at the end of 8 h, while G op exhibited a more sustained release pattern over the same period, where 70.29% ± 0.18 was released by 8 h (Figure 3A). Korsmeyer–Peppas seems to be the best mathematical kinetic model (R^2^ > 0.92) for gliclazide release from the tested beads (Figure 3B). A quadratic mathematical model was suggested for gliclazide release over 8 h (%DR 8 h) from the experimental factorial design of the study (Figure 3C–E). The significance of this model was indicated by a *p*-value of 0.0002, which was validated by an F-value of 373.31 (Figure 3F). Moreover, a small difference (<0.2) between predicted (R^2^ = 0.9873) and adjusted (R^2^= 0.9957) values, along with a high precision of 60.348 (>4 is statistically desirable), verified the accuracy and significance of the model (Figure 3G). Model parameters are denoted as follows: A: PDMS:TEOS ratio, B: alginate content, AB: the interaction between the PDMS:TEOS ratio and alginate content, A^2^: (PDMS:TEOS ratio)^2^, B^2^: (alginate content)^2^ (Figure 3F,H).

In this model, A, B, A^2^ and B^2^ were significant model terms (*p* value < 0.05) in determining the gliclazide release (%DR 8 h) from the formulated beads. However, AB was insignificant (*p*-value > 0.05) (Figure 3F). The relation between the actual and predicted values is presented in Figure 3I. The polynomial equation that governs %DR 8 h is % DR 8 h = 87.73 + 5.75 A − 7.53 B − 0.1607 AB − 2.84 A^2^ − 1.51 B^2^. Since AB is an insignificant model term, the equation can be simplified to % DR 8 h = 87.73 + 5.75 A − 7.53 B − 2.84 A^2^ − 1.51 B^2^. The two-dimensional contour plot between A and B indicated the non-linear relationship between these two variables in determining gliclazide release over 8 h (Figure 3J), while the three-dimensional plot revealed that gliclazide release (%DR 8 h) was decreased by increasing the alginate content and decreasing the PDMS:TEOS ratio (Figure 3K).

### 2.9. Optical Microscopy and Size Determination of the Beads

**Figure 4:**
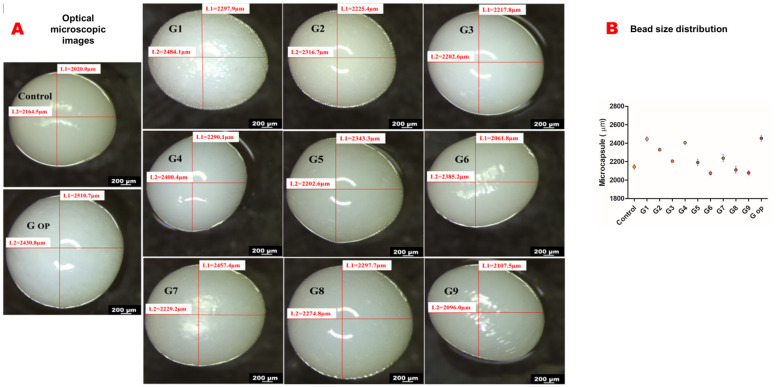
Optical microscopic images (scale bar 200 micrometres) (**A**). Bead size distribution (**B**).

All wet beads were spherical in shape with a smooth surface. However, G6 and G9 exhibited an oval shape. The blank control had the smallest size, while G op had the largest size of all formulations (Figure 4A,B).

### 2.10. Bead FTIR Study

**Figure 5:**
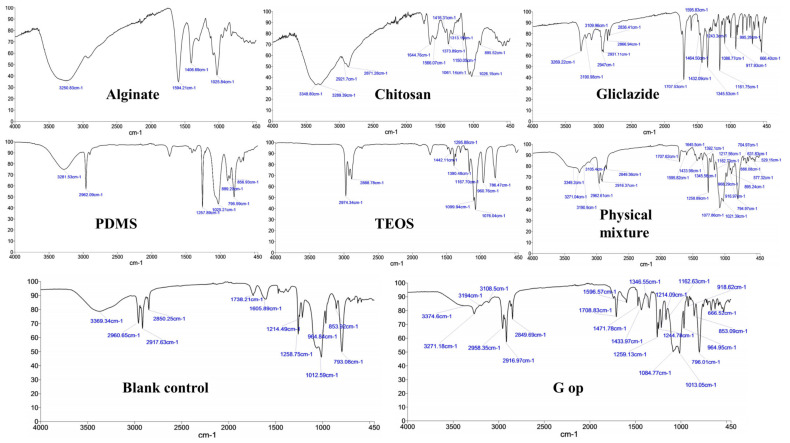
Characteristic FTIR bands/peaks in alginate, chitosan, gliclazide, PDMS, TEOS, physical mixture, blank empty control microcapsules and the optimised microcapsules (G op).

Characteristic peaks/bands for gliclazide [25,56,57], sodium alginate [25,56,57], PDMS [25,58,59], TEOS [25,60,61] and chitosan [38,62,63] are presented in Figure 5 and Table 2. Silanol (-Si-OH) peaks of PDMS at 3281.53 cm^−1^ and 889.2 cm^−1^ disappeared in both control and G op microcapsules due to the crosslinking of the polymer with TEOS as previously reported [25]. The broad peaks at 3250.83 cm^−1^ for alginate and chitosan peaks at 3348.8 cm^−1^ and 3289.39 cm^−1^ were replaced by a solo peak at 3369.34 cm^−1^ and 3374.6 cm^−1^ in the control and G op, respectively, with a remarkable decrease in the intensity, which suggests either an ionic interaction between -COO^−^ of alginate with -NH_3_^+^ of chitosan [64] or involvement of -OH of either chitosan or alginate in a condensation reaction with silanol terminals (-Si-OH) of hydrolysed TEOS [65]. Also, carboxylate peaks of alginate at 1594.21 cm^−1^ and 1406.69 cm^−1^ as well as the chitosan amide I peak (1644.76 cm^−1^) and amide II peak (1566.07 cm^−1^) were shifted to a different wavenumber (1605.89 cm^−1^ for control), which suggests either a complexation reaction between alginate and chitosan [64,66,67,68,69] or involvement of the chitosan amide group in an interaction with the silanol groups of TEOS [38]. Such an interaction could not be identified in G op due to the appearance of an -NH bending peak of gliclazide at 1596.57 cm^−1^ (Figure 5). FTIR of the physical mixture did not reveal any chemical interactions between used ingredients (Figure 5). Characteristic gliclazide peaks could be identified in the optimised formula (G op) among other excipients peaks, indicating the chemical stability and compatibility of the drug within the optimised formulation.

### 2.11. Bead Thermal Studies via DSC

**Figure 6:**
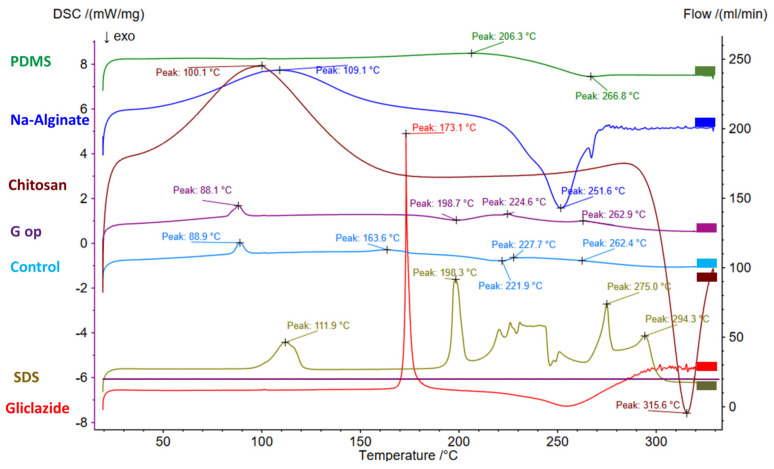
DSC thermograms in PDMS, alginate, chitosan, gliclazide, SDS, blank empty control microcapsules and the optimised microcapsules (G op).

DSC thermograms of gliclazide and the polymers employed in the beads (PDMS, alginate, chitosan) are presented in Figure 6 and Table 3. A sharp endothermic peak of gliclazide at 173.1 °C was identified that reflected the existence of the drug in its pure form. This peak was shifted to 163.6 °C in G op with a remarkable decrease in its intensity (Figure 6).

### 2.12. SEM/EDXR

**Figure 7:**
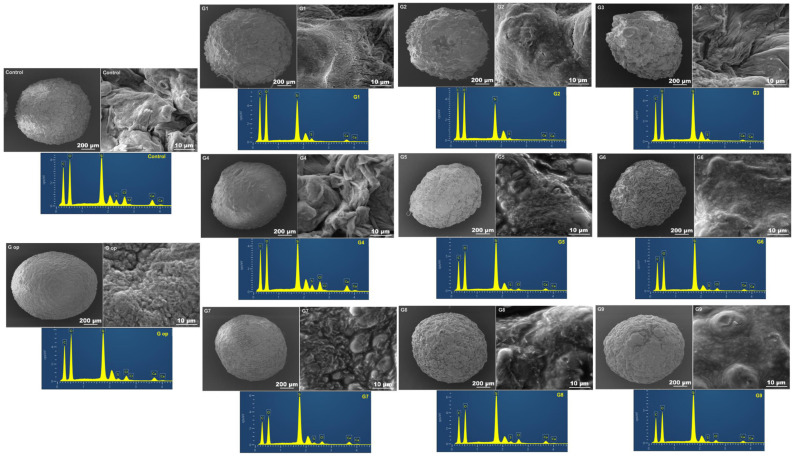
Scanning electron microscopic images of whole beads (scale bar 200 µm), bead surfaces (scale bar 10 µm) and EDXRs of bead surfaces.

The electron microscopic micrographs of all formulations showed compact, opaque, nonporous and discrete beads with rough to fibrous surfaces (Figure 7).

### 2.13. In Vitro Cell Viability Assay

**Figure 8:**
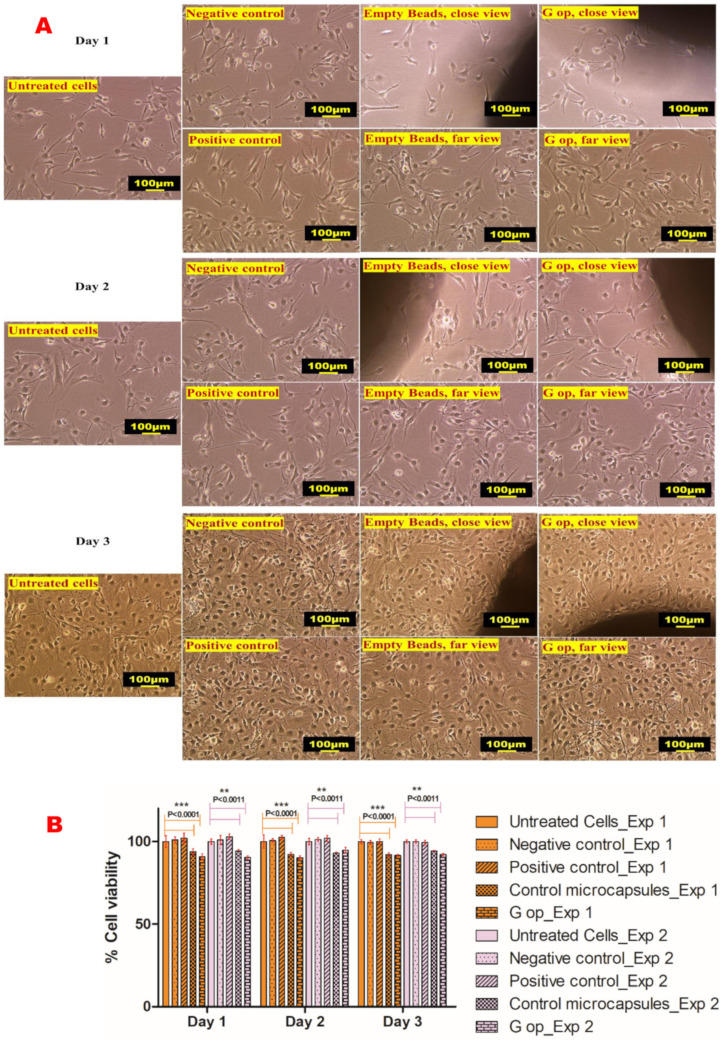
Cell viability results. Microphotographs of untreated C2C12 cells, negative control (DMSO-treated cells), positive control (gliclazide-treated cells) and treated cell lines with empty beads (control microcapsules) and the optimised formula (G op) (**A**). Percent cell viability results following 3-day exposure (representative data shown from two separate experiments) (**B**). Scale bar, 100 µm. * represents the degree of significance.

At the end of the 3-day exposure of cultured C2C12 myotubes, DMSO-treated cells (negative control) exhibited 99.3% ± 1.19 and 100% ± 1.02 cell viability, gliclazide-treated cells (positive control) exhibited 99.32% ± 1.56 and 100% ± 1.59 viability, empty control beads had 92.04% ± 1.19 and 94.22% ± 0.59 viability, while the cell viability for G op was 91.7% ± 0.59 and 92.17% ± 1.18 for the two separate experiments, indicating the biocompatibility of the novel beads with the C2C12 cell line (Figure 8).

## 3. Discussion

In this study, we have investigated grafting of a silicone elastomer (PDMS) crosslinked with an alkoxysilane (TEOS) at different ratios into the alginate backbone at different concentrations. A 3^2^ factorial design was employed through applying the response surface methodology to minimise the number of experimental attempts and to help find the optimised pharmaceutical formula [70,71]. A total of nine different experimental formulae were produced by varying the crosslinked silicone phase (first variable) and the Na-alginate (second variable) into which the silicone elastomer was grafted.

An ionic gelation process was employed to encapsulate the sulfonylurea-loaded polymeric blend in a crosslinker solution made of CaCl_2_, TEOS and chitosan to form free-flowing gliclazide beads. Early attempts to incorporate chitosan solution into the crosslinker bath resulted in very viscous media, which in turn produced irregular beads, especially due to the viscous nature of the gliclazide-loaded polymeric composite. A 0.5% *w*/*v* chitosan solution (dissolved in 1% acetic acid, two-step addition) was established as optimal for inclusion of chitosan into the crosslinking bath.

All formulations showed a non-linear shear thinning relationship (concaved downwards) between shear stress and shear rate. G op showed the highest recorded viscosity (resistance to flow) of all formulations, while G9 had the lowest viscosity at different shear rates (velocity gradients) in a non-Newtonian pseudoplastic fashion.

All gliclazide-loaded polymeric blends exhibited good electrokinetic stability of their surface charge (negative zeta potential). It seems that increasing the PDMS:TEOS ratio improved the electrokinetic stability of the developed drug-loaded vehicle. G8 formulated with PDMS:TEOS 4:1 showed the highest surface charge (more repulsive forces between particles), while G2 formulated with PDMS:TEOS 1:1 had the lowest surface charge (less repulsion). The positive charge of the crosslinking bath was attributed to the ionisation of chitosan amine terminals in the acidic pH of the crosslinking bath. Such a charge difference between the drug-loaded polymeric vehicle (negatively charged) and the crosslinker solution (positively charged) is anticipated to affect the bead size, shape or the dynamics of bead formation due to electrostatic attraction between opposite charges.

The improved gliclazide EE% by decreasing the PDMS:TEOS ratio and increasing the alginate content could be attributed to several factors, such as better crosslinking of PDMS with TEOS at closer ratios, the increased viscosity of the polymeric dispersion by increasing the alginate fraction which in turn minimised leaching of gliclazide during the ionic gelation process, possible additional film formation of chitosan–TEOS from the crosslinking media during the encapsulation process or due to better crosslinking of the alginate backbone with both Ca^++^ ions (egg box structure) and chitosan (opposite charge) in the crosslinking media. Furthermore, the acidic pH of the crosslinking bath itself retained more gliclazide within the beads due to low solubility of the sulfonylurea in this pH (less tendency of gliclazide to leach out of the beads) [72,73].

In acidic pH, all beads exhibited smaller swelling indices in these conditions compared to PBS, where the tested beads reached their maximum swelling in HCl at around 12 h, and thereafter less swelling was noticed until the end of the 24 h. This is due to the shrinkage of the alginate skeleton of the formulated beads resulting from the conversion of the water-soluble sodium alginate into the water-insoluble alginic acid in addition to the hydrophobic nature of the silicone elastomer scaffolded in the structure of the drug-loaded beads. G9 beads seem to be generally more permeable to aqueous uptake, as G9 had the highest swelling index of all formulations in both HCl and PBS. The silicone elastomer of G9 was crosslinked with TEOS at a 4:1 ratio, which might not be sufficient for a strong PDMS film that is resistant to the aqueous uptake of the tested media. It seems that G9 (with the lowest alginate content) in acidic media underwent less shrinkage, and together with a less crosslinked PDMS, the maximum swelling was reached in HCl. It has been reported, however, that the swelling of alginate/chitosan hydrogels increases with decreasing pH [64,74] due to solubilisation of the chitosan-bound fraction. Such an effect was not noticed in the formulated beads, probably due to involvement of chitosan in an interaction of interpenetrative film formation with TEOS [37,42] or due to the deposition of the inorganic silica (from the acid hydrolysed TEOS) within the bead core [75].

In PBS (pH 7.2), the swelling order of the tested beads was as follows: G9 > G6 > G3 > G8 > G5 > G2 > G7 > G4 > G op > G1 > blank control. It seems that at a fixed alginate content, formulations prepared by PDMS and crosslinked with TEOS at close ratios (1:1) have more hydrophobic properties (crosslinked better, less aqueous uptake) than those prepared at 2:1 and 4:1 ratios, due to the dense silica alginate bead core [75]. Also, at this pH, less swelling of the alginate/chitosan hydrogel is expected [64,74]. Swelling and loosening of the bead architecture in PBS is predominantly governed by sequestration of the Ca^++^ ions of the formulated bead’s skeleton (PDMS-grafted Ca-alginate) with Na^+^ ions of the buffer system. These effects resulted in intact yet swollen beads after 24 h.

The swelling properties of the tested formulations at pH 7.2 impacted the gliclazide release at a pH close to that of the dissolution media (pH 7.4). G9 and G6 beads that underwent much higher swelling in PBS released around 50% of their gliclazide payload in the first 3 h, similar to the marketed tablets. However, the least swollen formulations (G1, G op and G4) released the same gliclazide percentage over longer durations (5 h for G4 and 6 h for G1 and G op).

The retarded gliclazide release by increasing the alginate fraction and decreasing the PDMS:TEOS ratio identified by the three-dimensional presentation could be attributed to a stronger film formed by crosslinking the PDMS elastomer with TEOS at a closer ratio and retaining gliclazide within the bead structure. Another possible effect is the crosslinking of alginate with both Ca^++^ ions as well as chitosan that could have augmented the bead integrity by forming a denser bead structure with a compact core. Of importance, Gaserod et al. reported that chitosan exists in a less extended polymeric conformation in higher ionic strength of Ca^++^ ions, which facilitates its diffusion from the crosslinking bath into the alginate bead core that binds to it at a faster rate and to a greater extent [29,30].

To understand the drug release mechanism from the formulated beads, the in vitro gliclazide release curves were fit into different mathematical kinetic models (zero order, first order, Higuchi and Korsmeyer–Peppas). Correlation coefficients (R^2^) of the tested beads seemed to follow the Korsmeyer–Peppas kinetic model (R^2^ ranged between 0.924 and 0.952), indicating a controlled release mechanism. This sustained gliclazide release from the composite beads is thought to be due to the formation of an intertwined scaffold network between the hydrophilic organic polysaccharide (alginate) and the hydrophobic inorganic crosslinked silicone elastomer through which gliclazide diffuses via the swollen carbohydrate segments, followed by formation of the solid gliclazide/gelled polysaccharide boundary and eventually a concentration gradient diffusion of gliclazide from composite beads into the dissolution media [76].

The diffusional exponent (n) from the Korsmeyer–Peppas kinetic model varied and ranged between 0.770 and 2.339. G3, G5, G6, G8 and G9 exhibited non-Fickian (anomalous) transport (1 > *n* > 0.5) due to a combined effect of polymeric matrix dissolution and drug diffusion [76,77]. The release curves of these formulations showed a good fit with the first-order model, too, identified by a high (R^2^) value which indicated that the drug release from their matrices is dependent on the initial gliclazide concentration [78]. However, G1, G2, G4, G7 and G op exhibited super case II transport (*n* > 1) governed by swelling and relaxation of bead architecture, probably due to disentanglement and erosion of the polymeric scaffold [76,78,79].

G1, G2, G3 formulated with PDMS:TEOS at a 1:1 ratio have more spherical shapes with relatively bigger bead sizes than others, as shown by light microscopy. This could be attributed to the lower zeta potential (less negative charge) they possess compared to the other formulations (less electrostatic attraction with the crosslinker). G1 had the highest viscosity profile of all nine formulations. The round to oval shape of G6 and G9 could be attributed to their lower alginate content in addition to the low viscosity that their polymeric dispersions exhibited. G6 and G9 had high zeta potentials of −104.3 mV± 3.22 and −102 mV ± 3.23, respectively, which could have contributed to the viscosities of their polymeric dispersion in determining the shape and size of the produced beads. On the other hand, G op exhibited a larger bead size with a more regular spherical shape, probably due to the high viscosity of its polymeric dispersion with respect to its zeta potential.

Characteristic gliclazide FTIR peaks could be identified in the optimised formula (G op), while its sharp endothermic melting point at 173.1 °C (due to its pure crystalline state) was shifted to 163.6 °C in G op with a remarkable decrease in intensity. Such a change to the physical characteristics of gliclazide could be attributed to its dispersion within the bead skeleton, its conversion to the non-crystalline form or its inclusion within beads at the molecular level [25,80]. Also, the endothermic broad peaks of alginate (109.1 °C) and chitosan (100.1 °C) indicated that residual water evaporation [81] was shifted to lower temperatures (88.1 °C in control and 88.9 °C in G op). This shift in the residual water elimination temperature was proposed to be due to less water retention by the less hydrophilic crosslinked composite bead surface [81].

The scanning electron micrographs revealed the spherical shapes of all formulated composite beads, even after vacuuming and drying, due to the elastomeric properties of the silicone skeleton in preserving the original spherical shape of the ionically gelled crosslinked beads. All beads exhibited continuous opaque surfaces. G7, G8 and G9 formulated with PDMS:TEOS at a 4:1 ratio appeared with more surface bulges, probably due to dissociation of PDMS (less crosslinked) from the intertwined polymeric network. Likewise, G5 and G6 formulated with PDMS:TEOS at a 2:1 ratio exhibited fewer surface bulges than G7, G8 and G9. Control, G1, G2, G3 and G4 beads exhibited rough to fibrous surfaces. However, G op had much smoother bulge-free appearance with a wavey to serrated surface. EDXR revealed an even distribution of the carbohydrate polymers (alginate and chitosan) identified by oxygen (O), carbon (C), PDMS and the alkoxysilane TEOS (Si). Sulphur (S) could also be identified on the bead surface, which is probably due to SDS (employed in PDMS emulsification and the crosslinking process) rather than gliclazide, where nitrogen (N), a characteristic atom of gliclazide, could not be detected at the bead surface, which suggests the encapsulation/entrapment of gliclazide within the bead core. Nitrogen is also characteristic for chitosan biopolymers, and its disappearance from the bead surface suggests either chitosan diffusion within the bead core as outlined by Gaserod et al. [29,30], or possibly the involvement of the cationic -NH3^+^ of chitosan with either the anionic -COO^−^ of alginate or with silica of TEOS to form an interpenetrating networked bead structure via crosslinking the polymeric blend with CaCl_2_, chitosan and TEOS of the crosslinking bath rather than coating the beads with chitosan and TEOS.

In vitro cytotoxicity studies of the formulated microcapsules revealed their biosafety and biocompatibility in cultured C2C12 cell lines. A few reports have raised some concerns about the chemical and biological inertness of some siloxane-based formulations [82,83,84], where fibrotic reactions, tissue inflammation and fibromyalgia were reported with the use of some implantable silicones without adequate explanation [82,83,84]. Recently, some PDMS-based coatings showed cytotoxicity to the mouse fibroblast L929 and the hamster lung fibroblast V79 cell lines in a concentration-dependent manner [85]. Despite multiple reports on their safety [86,87,88,89], a few studies have revealed the cytotoxicity of some siloxanes in human cultured cells [90] and human lymphocytes [91]. These conflicting data about the biosafety of PDMS necessitate the in vitro testing of silicone-based formulations or inserts prior to their clinical application or even animal trials. In this study, our novel gliclazide beads showed promising pharmaceutical suitability and sustained release behaviour in addition to their biocompatibility with skeletal myoblasts, and suggest their safe application in an implantable form to target their payload via the skeletal muscle delivery route.

## 4. Materials and Methods

Chitosan (CAS 9012764, 50,000–190,000 Da), sodium dodecyl sulfate, SDS (CAS 151213), alginic acid sodium salt (CAS 9005383, low viscosity), tetraethyl orthosilicate, TEOS (CAS 78104), polydimethylsiloxane (hydroxy terminated PDMS, CAS 70131678) and dimethyl sulfoxide (DMSO, CAS 67685) were obtained from Sigma-Aldrich (St Louis, MO, USA). Calcium chloride (96%, anhydrous) and gliclazide (99.9%, CAS 21187984) were procured from ThermoFisher Scientific (Melbourne, Australia). The mouse myoblast C2C12 cell line was obtained from the American Tissue Culture Collection (ATCC; Gaithersburg, MD, USA). The rest of the chemicals and reagents were of HPLC grade.

### 4.1. Microcapsule Preparation

**Figure 9:**
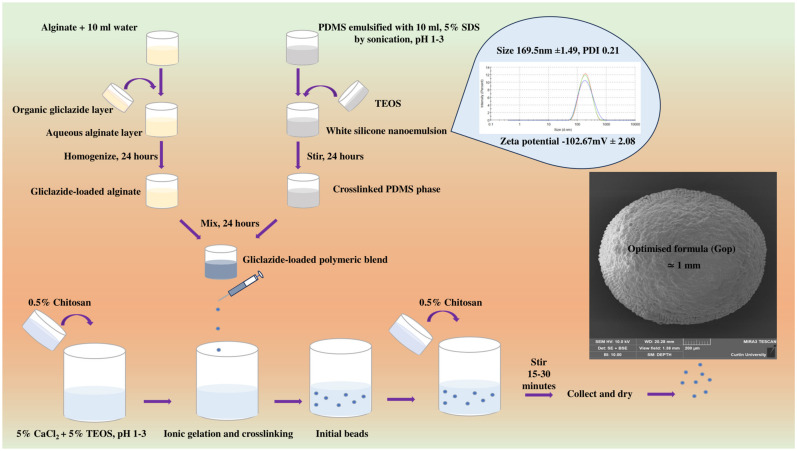
Schematic of formulation procedure.

Using a Bransonic ultrasonic bath (Danbury, CT, USA), PDMS was emulsified in acidified 5% SDS (pH 1–3) for 15 min followed by sonication using a UP200S probe (Teltow, Germany) for an additional 1 minute until a white elastomeric nanoemulsion was formed. This nanoemulsion was crosslinked by addition of TEOS as per the specified ratio in Table 1 and stirred for 24 h (for adequate crosslinking). Concurrently, gliclazide was dissolved in tetrahydrofuran/dichloromethane/hexane at a 1:1:1 ratio and homogenised with aqueous sodium alginate for 24 h. Silicone latex and gliclazide-loaded alginate were combined and further stirred for 24 h. The polymer to drug ratio was maintained at 2:1 in all formulations. Gliclazide-loaded polymeric composite was extruded at 10–15 cm height using a 25 G needle into a gently agitated crosslinking bath (pH 1–3) made of 5% CaCl_2_, 5% TEOS and 0.5% chitosan solution. Half of the chitosan solution (50 mL) was added before the encapsulation process and the second half (50 mL) was added at the end of the encapsulation process. Control microcapsules (empty, blank) were formulated the same way without the inclusion of gliclazide. All formulated beads were left in the crosslinking bath for 15–30 for completion of the ionic gelation process and the polyelectrolyte coacervate formation. Beads were collected by decantation and washed thrice with deionised water, dried at 37 °C for a week and stored in a desiccator for further studies (Table 1) (Figure 9).

### 4.2. Experimental Design

Using Design-Expert^®^ 13 software (Stat-Ease Inc., Minneapolis, MN, USA), a 3^2^ factorial design was utilised to formulate 9 different experimental microcapsules by varying two different parameters (PDMS:TEOS ratio and Na-alginate content) at 3 different levels (low, medium and high) as per Table 1. Encapsulation efficiency (EE%) and cumulative drug release at 8 h (%DR 8 h) were considered as dependent responses in the optimisation process by response surface methodology and comparing the estimated results based on suggested conditions by Design-Expert^®^ 13 software and the practical values obtained from the experimental work (Table 1).

### 4.3. Pre-Encapsulation Assessment of Gliclazide-Loaded Silicone-Grafted Alginate

#### 4.3.1. Rheological Properties

The rheological parameters, shear stress, shear rate and viscosity of drug-loaded polymeric vehicles of all formulae were determined at different speeds at room temperature using a Bohlin Visco 88 viscometer (Worcestershire, UK) and presented as the mean ± SD (*n* = 3). The viscometer spindle was immersed in a sample holder containing around 5 mL of tested sample and allowed to freely rotate at different viscometer speeds at room temperature.

#### 4.3.2. Electrokinetic Stability

A Zetasizer Nano ZSP (Malvern, UK) was employed to measure the zeta potential of the drug-loaded polymeric vehicles of all formulations as well as the crosslinking bath (before and after the encapsulation process) by diluting 5 drops of every sample in 5 mL deionised water using a suitable cuvette as per lab protocol. Data are presented as mean ± SD (*n* = 3).

#### 4.3.3. Characterisation of the Silicone Latex (Nanoemulsion) Employed in the Optimised Formula (G op)

A Zetasizer Nano ZSP (Malvern, UK) was employed to measure the zeta potential and particle size of PDMS emulsified with SDS by the dynamic light scattering technology by diluting 5 drops of every sample in 5 ml deionised water using a suitable cuvette (sample holder) as per lab protocol. Data are presented as mean ± SD (*n* = 3).

### 4.4. Post-Encapsulation Assessment of Gliclazide-Loaded Beads

#### 4.4.1. Flow Properties and Compressibility of Microcapsules

In a glass cylinder, the volume occupied by two grams of every formulation before and after tapping of the glass cylinder 100 times on the lab bench was used to determine the bulk density and tapped density, respectively. Average determination (*n* = 3, ±SD) of Carr’s index (compressibility index) and Hausner’s ratio are calculated in Equations (1) and (2), respectively [25].

(1) $$Carr’s\;index\;\%=\frac{Density\;\left(tapped\right)-Density\;(bulk)}{Density\;(tapped)}\times 100$$

(2) $$Hausner’s\;ratio=\frac{Density\;(tapped)}{Density\;(bulk)}$$

#### 4.4.2. Entrapment Efficiency and Gliclazide Loading/Content

For each formulation, 100 mg of the beads were ground and shaken for 24 h in 100 mL PBS (pH 7.4) at 37 °C using a multishaker (PSU 20, 150 rpm) (Melbourne, Australia). Samples were centrifuged and filtered with a 0.45 µm syringe filter for HPLC determination of gliclazide using a previously reported HPLC method at 227 nm [56,57]. Gliclazide content (GL%) and entrapment efficiency (EE%) were calculated and presented as mean ± SD (*n* = 3) by Equations (3) and (4), respectively [24,25].

(3) $$GL\%=\frac{Gliclazide\;content\;in\;microcapsule\;sample}{Microcapsule\;sample\;weight}\times 100$$

(4) $$EE\%=\frac{Observed\;weight\;of\;gliclazide}{Theoretical\;weight\;of\;gliclazide}\times 100$$

#### 4.4.3. Microcapsule Mechanical Integrity

Every formula was tested in two different media (0.9% NaCl saline solution and PBS, pH 6.8) in triplicate. Fifteen microcapsules of every formula were placed in a 50 mL flask of tested media and shaken for 24 h at 37 °C using a multishaker (PSU 20, 150 rpm) (Melbourne, Australia) [24,25]. Visual counting of intact microcapsules after shaking was used to determine the mechanical integrity of microcapsules using Equation (5):

(5) $$Microcapsule’s\;mechanical\;integrity=\frac{Microcapsule\;count\;after\;shaking}{Microcapsule\;count\;before\;shaking}\times 100$$

#### 4.4.4. Swelling Behaviour of Microcapsules

Swelling studies of every formula were conducted in two different media (0.1N HCl, pH 1.2 and PBS, pH 7.2) in triplicate (*n* = 3, ±SD). In a dissolution basket and using an Erweka DT6 dissolution apparatus (Heusenstamm, Germany) at 37 °C, 100 mg of every formula was tested in the selected media without rotation. Swollen beads were collected at predetermined timepoints over 24 h, and surface droplets were removed by a paper towel. The swelling index was used to calculate the relation between the weight of initial dry beads and their weight after swelling [24,25], as per Equation (6):

(6) $$Swelling\;index\;\%=\frac{Weight\;of\;swollen\;microcapsules-initial\;weight\;of\;dry\;microcapsules}{Initial\;weight\;of\;dry\;microcapsules}\times 100$$

#### 4.4.5. In Vitro Gliclazide Release and Release Kinetics

The gliclazide release from different microcapsules was determined in PBS, pH 7.4 at 37 °C using an Erweka DT6 dissolution apparatus (Heusenstamm, Germany) (900 mL, paddle, 100 rpm). A sample microcapsule (equivalent to 100 mg gliclazide) was placed in the dissolution apparatus vessel (*n* = 3). An aliquot of 5 mL was withdrawn from each vessel and filtered using a 0.45 µm syringe filter for HPLC determination using a previously reported HPLC method at 227 nm [92,93]. To maintain sink conditions, 5 mL of fresh PBS, pH 7.4 were added to replace the sampled volume. The drug release from a commercial modified release gliclazide formula (Diamicron^®^ 30MR tablet) was tested for comparison of the cumulative drug release, and data are presented as mean ± SD (*n* = 3). The dissolution profile of every formulation was kinetically evaluated by applying various mathematical models, namely zero-order, first-order, Higuchi and Korsmeyer–Peppas kinetic models. The best fit was chosen based on the correlation coefficient (R^2^) from the regression analysis performed via a Microsoft Excel, version 2402, Washington, DC, USA add-in, DD solver [94].

#### 4.4.6. Optical Microscopy and Bead Size Determination

The surface morphology of wet microcapsules from each formula was investigated against a dark background by a Nikon SMZ800 stereo optical microscope (Nikon, Melville, NY, USA) fitted with a Toupcam 14 MPA camera. The microcapsule diameter (*n* = 5, ±SD) of each formula was measured with Toupview software, version 3.7 (Melville, NY, USA) attached to the optical microscope.

#### 4.4.7. Fourier Transform Infrared Spectroscopy (FTIR)

A Perkin Elmer (Waltham, MA, USA) spectrophotometer was used to study the drug excipient compatibility by scanning gliclazide, TEOS, PDMS, sodium alginate, chitosan, physical mixture, blank control microcapsules and the optimised formula (G op) in the range of 4000 cm^−1^ to 450 cm^−1^.

#### 4.4.8. Differential Scanning Calorimetry (DSC)

A NETZSCH DSC 3500 (Sirius, Hamburg, Germany) was used to record the thermograms of sodium alginate, chitosan, PDMS, gliclazide, SDS, blank control microcapsules and the optimised formula (G op) under a 20 mL/min nitrogen purge. A total of 5 mg of each sample in a sealed aluminium pan (compared to control empty aluminium pan) was ramp-heated at a 20 °C/min rate from 20 °C to 350 °C.

#### 4.4.9. Electron Microscopy (SEM) and Energy Dispersive X-ray (EDXR)

A sample of a few microcapsules from every formula was placed on a suitable glass stub and coated with platinum. An electron beam of 5 kV emitted from a Tescan MIRA3 XMU electron microscope (Brno, Czech Republic) connected to an EDX, Oxford X-MAX^N^ 150 SDD X-ray detector and fitted with Aztec software, version 6.1 (Oxford Instruments, Abingdon, UK) was used to capture and record the electron micrographs and the topographic features of the tested beads.

#### 4.4.10. Cell Culture and Viability Assay

The C2C12 cell line was cultured in T75 flasks in high glucose Dulbecco’s Modified Eagle’s Medium (DMEM, Sigma Aldrich) supplemented with 10% bovine serum and 1% streptomycin/penicillin solution in a NuAire^®^ incubator (5% CO_2_, 37 °C, 95% humidity). The culturing medium was replaced every two days until cells reached 80% confluency. C2C12 cells were subcultured in T25 flasks and seeded at a density of 2 × 10^5^ cells/T25 flask and incubated for 24 h to form a monolayer of the myoblast cells. On two different occasions and in triplicate, C2C12 cells were exposed to DMSO (negative control), gliclazide (2 mol/L) dissolved in DMSO (positive control) [47], drug-free control microcapsules (empty microcapsules) and the optimised formula (G op). Direct contact of tested beads with C2C12 was assessed as the main exposure method (by placing sample beads in the middle of a T25 flask) as per in vitro cytotoxicity and biological evaluation of medical devices ISO 10993-5:2009 [95] and quality control of biomaterials [60]. Cell viability was determined by microscopic examination of live cells (in terms of membrane integrity, morphology and count) [96] for 3 consecutive days, and percent cell viability was calculated as per Equation (7) using a Nikon Eclipse Ts 100 microscope fitted with Nikon image capturing screen.

(7) $$Cell\;viability\;\%=\frac{Average\;number\;of\;live\;cells\;(treated)}{Average\;number\;of\;live\;cells\;(untreated)}\times 100$$

### 4.5. Statistical Analysis

Design Expert^®^ 13 software (Stat-Ease Inc., Minneapolis, MN, USA) and GraphPad Prism software (GraphPad Inc., version 5, San Diego, CA, USA) were used for graphical presentation and the statistical analysis of mean ± SD triplicate data using raw means/totals and one-way ANOVA, and a *p*-value < 0.05 was considered as statistically significant.

## 5. Conclusions

The current study reported the development and characterisation of novel controlled-release organic–inorganic elastomeric composite beads loaded with gliclazide. A 3^2^ experimental factorial design was employed to optimise the pharmaceutical formula based on selection criteria of the highest gliclazide content that is released over a longer duration. The quadratic mathematic model and the 3D plot suggested for both encapsulation efficiency and gliclazide release that the silicone elastomer crosslinked at a 1:1 ratio, and the higher alginate content is required to obtain higher gliclazide encapsulation efficiency and to sustain the release of the contained sulfonylurea. These data are linked to the swelling profile of the novel beads (remained intact after 24 h in PBS), where the optimised formula (G op) exhibited controlled release of gliclazide with case II transport (*n* > 1) governed by swelling and followed by relaxation of the bead architecture to sustain the release of the contained sulfonylurea that follows the Korsmeyer–Peppas release kinetic model. The DSC data revealed a change in the physicochemical properties of encapsulated gliclazide without affecting its chemical structure (identified by FTIR). G op had an opaque round shape, visualised by SEM, with a wavey serrated smooth surface. In vitro testing of the novel platform on C2C12 myoblasts revealed their biosafety and biocompatibility, and suggest their application as an implantable drug delivery vehicle that could be targeted to the skeletal muscle while bypassing the hepatic metabolism of pharmacotherapeutics of interest. The in vitro cell viability results of this novel biomaterial scaffold warrant further investigation in future studies.

## Figures and Tables

**Table 1:** Composition of each formula, encapsulation efficiency (EE%), gliclazide release over 8 h (%DR 8 h), Carr’s index and Hausner ratio.

Formula Code	PDMS: TEOS (g: g)	Alginate (g)	EE% (Mean ± SD, *n* = 3)	%DR 8 h (Mean ± SD, *n* = 3)	Carr’s Index (Mean ± SD, *n* = 3)	Hausner Ratio (Mean ± SD, *n* = 3)
Control	0.8: 0.8	0.4	-	-	11.07 ± 1.28	1.12 ± 0.016
G1	0.8: 0.8	0.4	93.20 ± 0.24	70.56 ± 0.36	12.29 ± 1.21	1.14 ± 0.015
G2	0.8: 0.8	0.3	86.29 ± 0.13	78.79 ± 0.82	11.16 ± 2.25	1.13 ± 0.028
G3	0.8: 0.8	0.2	80.18 ± 0.19	85.04 ± 0.19	11.89 ± 0.49	1.14 ± 0.006
G4	0.8: 0.4	0.4	88.03 ± 0.31	76.03 ± 0.60	12.18 ± 0.88	1.14 ± 0.011
G5	0.8: 0.4	0.3	80.93 ± 0.60	86.07 ± 0.15	11.05 ± 1.59	1.12 ± 0.020
G6	0.8: 0.4	0.2	75.15 ± 0.18	91.36 ± 0.11	11.66 ± 1.58	1.13 ± 0.021
G7	0.8: 0.2	0.4	82.88 ± 0.33	81.62 ± 0.83	11.82 ± 1.13	1.13 ± 0.014
G8	0.8: 0.2	0.3	76.01 ± 0.24	90.41 ± 0.59	11.75 ± 1.33	1.13 ± 0.017
G9	0.8: 0.2	0.2	72.32 ± 0.47	96.87 ± 0.16	11.90 ± 0.57	1.14 ± 0.007
G op	0.8: 0.437	0.493	96.98 Predicted/estimated	64.55 Predicted/estimated	-	-
G op (Practical)			93.48 ± 0.19 Observed/practical	70.29 ± 0.18 Observed/practical	12.21 ± 0.31	1.14 ± 0.004
$\%\;\mathrm{Error}=\frac{Practical\;value\;-\;Estimated\;value}{Estimated\;value}\times 100$			−3.61%	8.89%	-	-

**Table 2:** Characteristic FTIR bands/peaks of Na-alginate, chitosan, PDMS, TEOS and gliclazide.

Ingredient	Functional Group	Appeared as FTIR Peak/Band at	Reference
Na-alginate	-OH stretching vibration from carboxyl group	3250.83 cm^−1^ (broad peak)	[25,56,57]
	Carboxylate stretching vibration, asymmetric	1594.21 cm^−1^	
	Carboxylate stretching vibration, symmetric	1406.69 cm^−1^	
	stretching vibration (-C-O-C-)	1025.84 cm^−1^	
Chitosan	-OH stretching	3348.80 cm^−1^	[38,62,63]
	-NH stretching	3289.39 cm^−1^	
	Aliphatic -CH stretching	2921.7 cm^−1^, 2871.28 cm^−1^	
	Amide I band (-C=O stretching)	1644.76 cm^−1^	
	Amide II band (bending vibration/deformation of -NH_2_)	1566.07 cm^−1^	
	-CH bending/wagging	1416.31 cm^−1^	
	-OH bending	1373.89 cm^−1^	
	Amide III band (-CN stretching)	1313.19 cm^−1^	
	Asymmetric stretching vibration of glycosidic linkage (-C-O-C-)	1150.05 cm^−1^	
	Skeletal vibration involving (-CO stretching)	1061.14 cm^−1^ and 1026.15 cm^−1^	
	Related to saccharide structure	895.52 cm^−1^	
PDMS elastomer	Silanol (-Si-OH)	3281.53 cm^−1^, broad peak	[25,58,59]
	(-Si-CH_3_)	2962.09 cm^−1^, sharp peak	
	(-Si (CH_3_)_2_), symmetric stretching	1257.89 cm^−1^, sharp peak	
	(-Si-O-Si-), asymmetric stretching	1025.21 cm^−1^	
	Silanol (-Si-OH)	889.2 cm^−1^	
	(-Si-CH_3_)	856.93 cm^−1^	
	Asymmetric bending (-Si-(CH_3_)_2_)	795.59 cm^−1^, sharp	
TEOS	Aliphatic (-CH stretching) in the ester group	2974.34 cm^−1^ and 2888.78 cm^−1^	[25,60,61]
	Asymmetric wagging/bending (-CH)	Small peaks at 1442.11 cm^−1^, 1390.48 cm^−1^ and 1295.88 cm^−1^	
	CH_3_ rocking	1167.7 cm^−1^	
	Asymmetric stretching of Si attached ethoxy group (Si-O-C-O-)	1099.94 cm^−1^	
	-CH rocking	960.76 cm^−1^	
Gliclazide	-NH	3269.22 cm^−1^	[25,56,57]
	-CH, aromatic	3190.98 cm^−1^ and 3109.96 cm^−1^	
	Aliphatic perhydro-cyclopenta pyrrole ring (-CH stretching)	Peaks in the range 2947 cm^−1^–2836.41 cm^−1^	
	Carbonyl stretch -C=O	1707.53 cm^−1^, sharp peak	
	-NH bending	1595.83 cm^−1^	
	C=C stretching, aromatic	1432.09 cm^−1^	
	Sulfonyl stretching (-S=O), asymmetric	1345.53 cm^−1^	
	Heterocyclic C-N ring stretch	1243.3 cm^−1^	
	Sulfonyl vibration(-S=O), symmetric	1161.75 cm^−1^	
	-C-O stretching	1086.77 cm^−1^	
	C=C bending	995.26 cm^−1^	
	Phenyl, aromatic P substitution	917.93 cm^−1^	
	Aromatic ring	666.40 cm^−1^	

**Table 3:** DSC thermograms of PDMS, SDS, Na-alginate, chitosan, gliclazide, empty control microcapsules and optimised microcapsules (G op).

Ingredient	DSC Thermogram Peaks
PDMS	Endothermic peak at 206.3 °C Exothermic peak at 266.8 °C
SDS	Early endothermic peak at 111.9 °C (dehydration) Sharp endothermic melting peak at 198.3 °C Late peaks at 275 °C and 294.3 °C (decomposition)
Na-alginate	Broad endothermic peak at 109.1 °C (dehydration) Exothermic peak at 251.6 °C (polymer decomposition)
Chitosan	Broad endothermic peak at 100.1 °C (dehydration) Exothermic peak at 315.6 °C (polymer decomposition)
Gliclazide	Sharp endothermic melting peak at 173.1 °C
Control microcapsule	Endothermic peaks at 88.1 °C, 224.6 °C and 262.9 °C Exothermic peak at 198.7 °C
G op	Endothermic peaks at 88.9 °C, 163.6 °C, 227.7 °C and 262.4 °C Exothermic peak at 221.9 °C

## Data Availability

Data is contained within the article.
